# Supplementary figures and images for: Lens Epithelial Explants Treated with Vitreous Humor Undergo Alterations in Chromatin Landscape with Concurrent Activation of Genes Associated with Fiber Cell Differentiation and Innate Immune Response
Source: Cells. 2023 Feb 3;12(3):501. doi: 10.3390/cells12030501 (PMC9914805; doi:10.3390/cells12030501)

A.

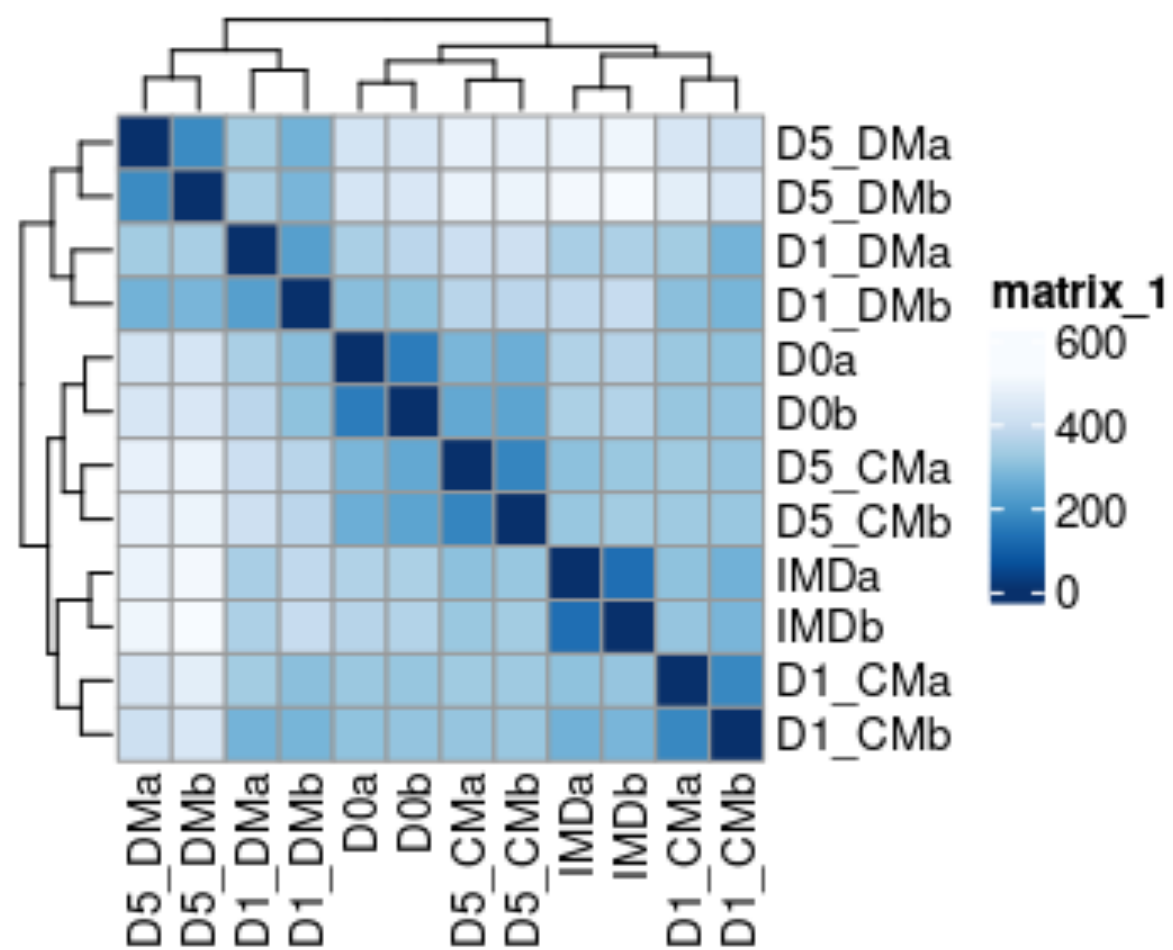

B.

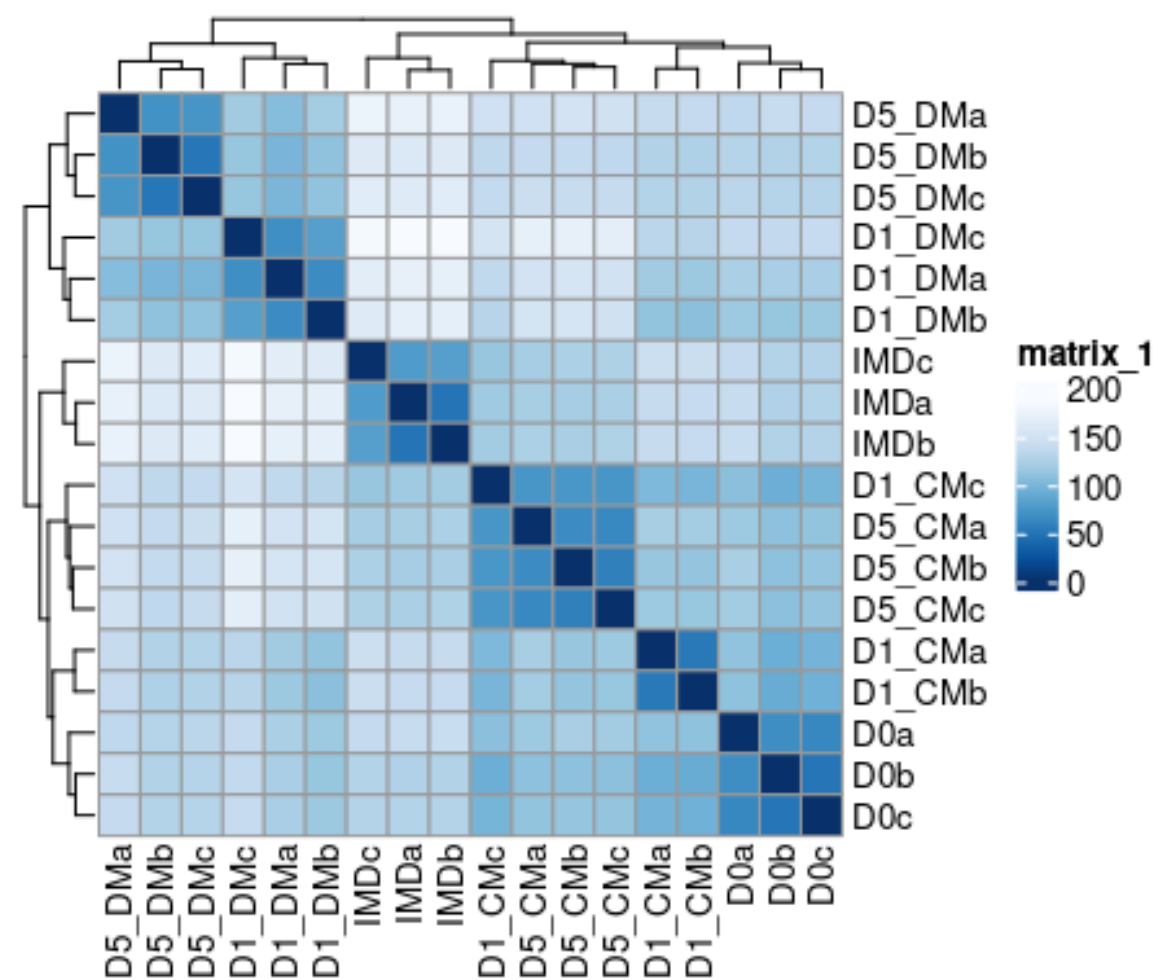

Supplement: Supplementary file 1 [file cells-12-00501-s001.zip › Figure_S1.pdf]

A

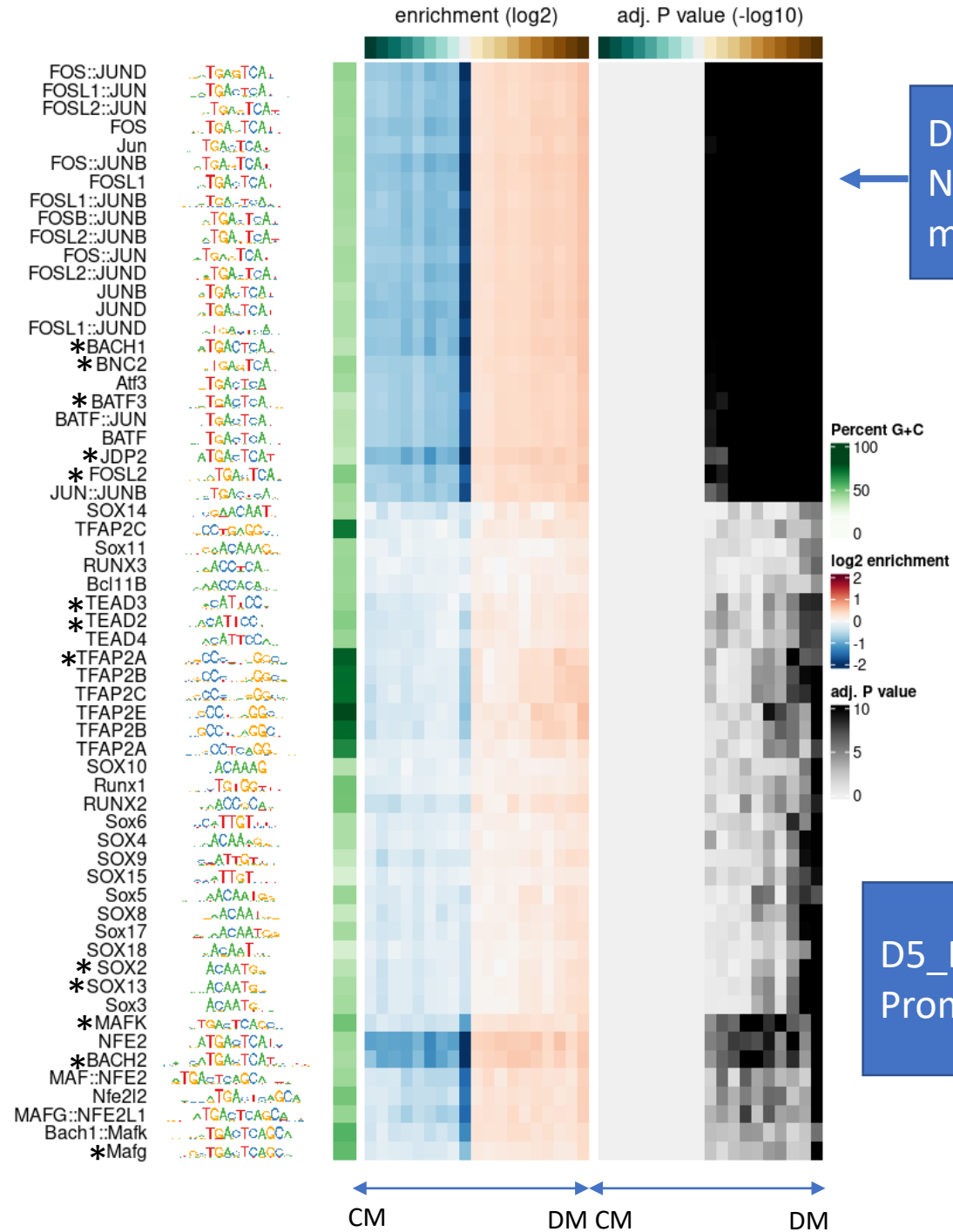

B.

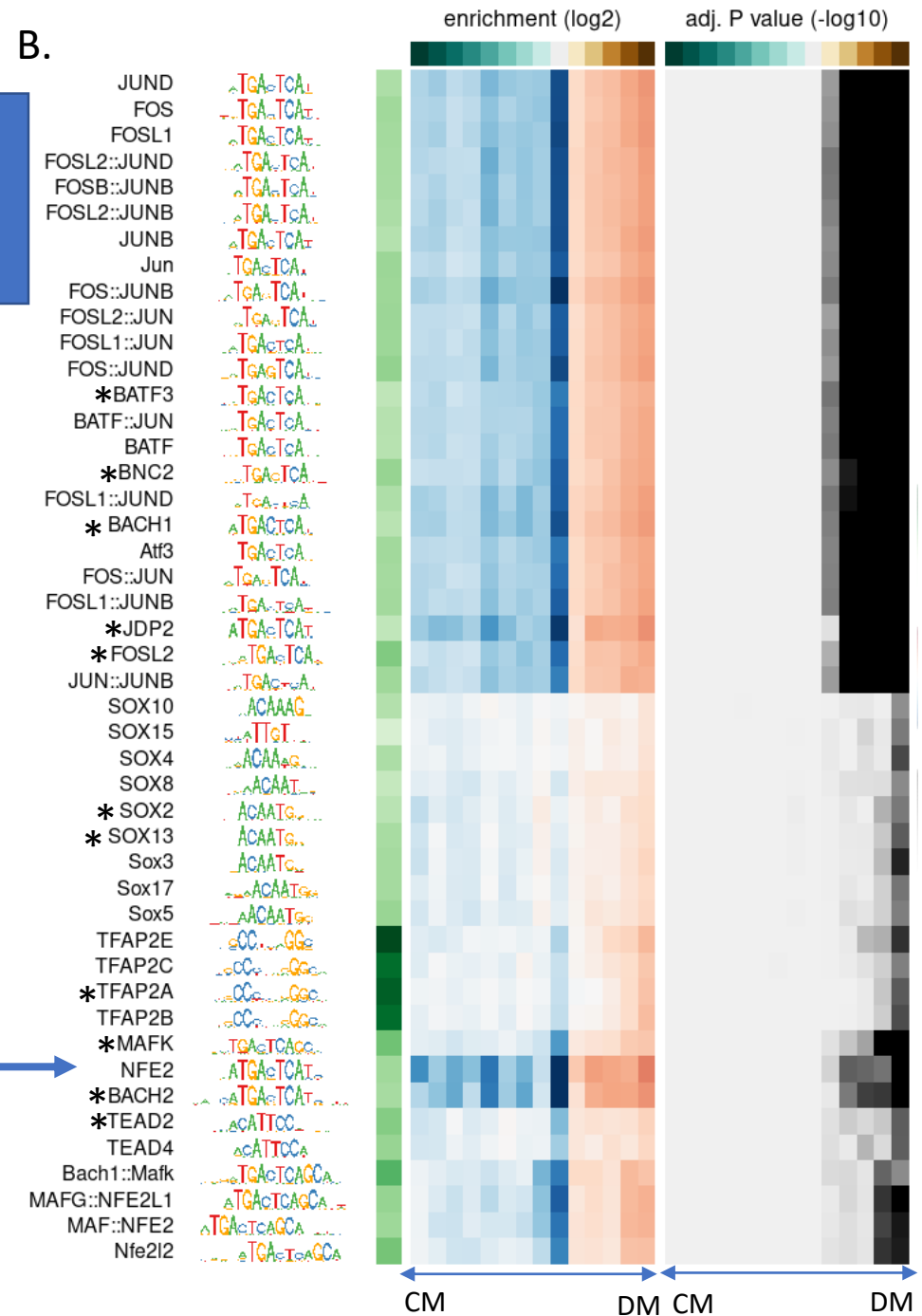

Supplement: Supplementary file 1 [file cells-12-00501-s001.zip › Figure_S10.pdf]

A.

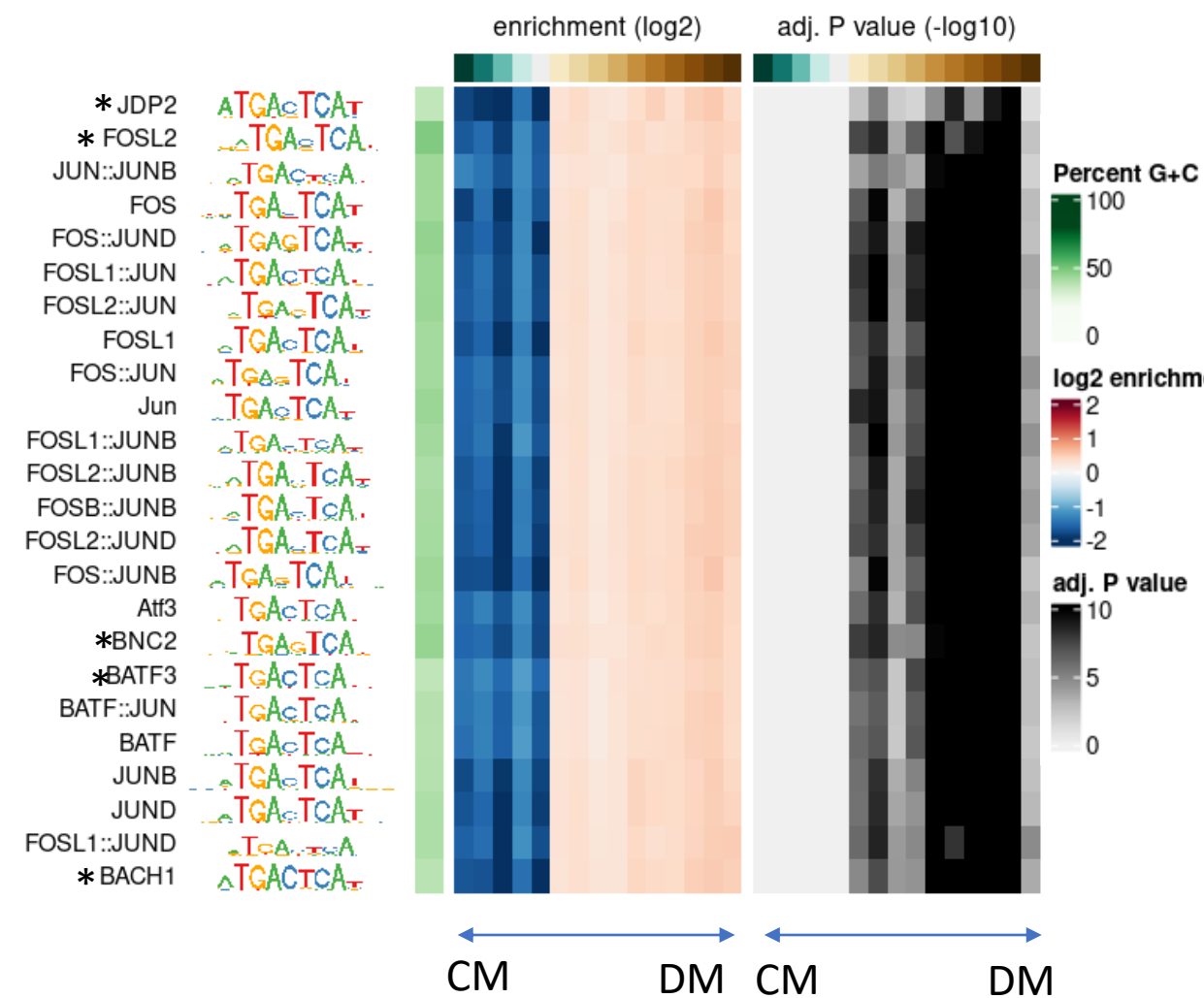

D1\_DM\_Non-promoter motif

B.

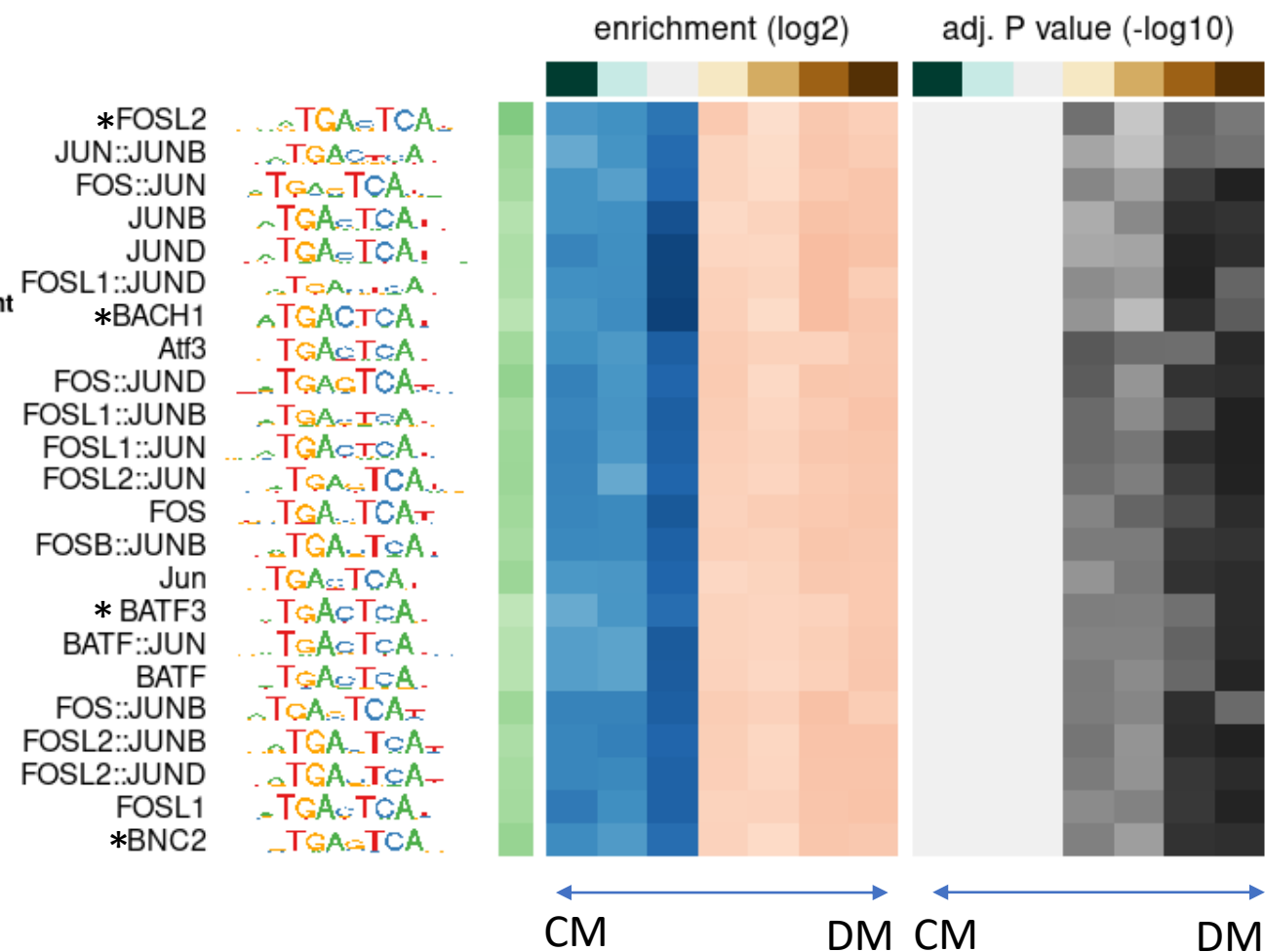

D1\_DM\_Promoter motif

Supplement: Supplementary file 1 [file cells-12-00501-s001.zip › Figure_S11.pdf]

**D5\_DM\_Promoter**

**D1\_DM\_Non-Promot**

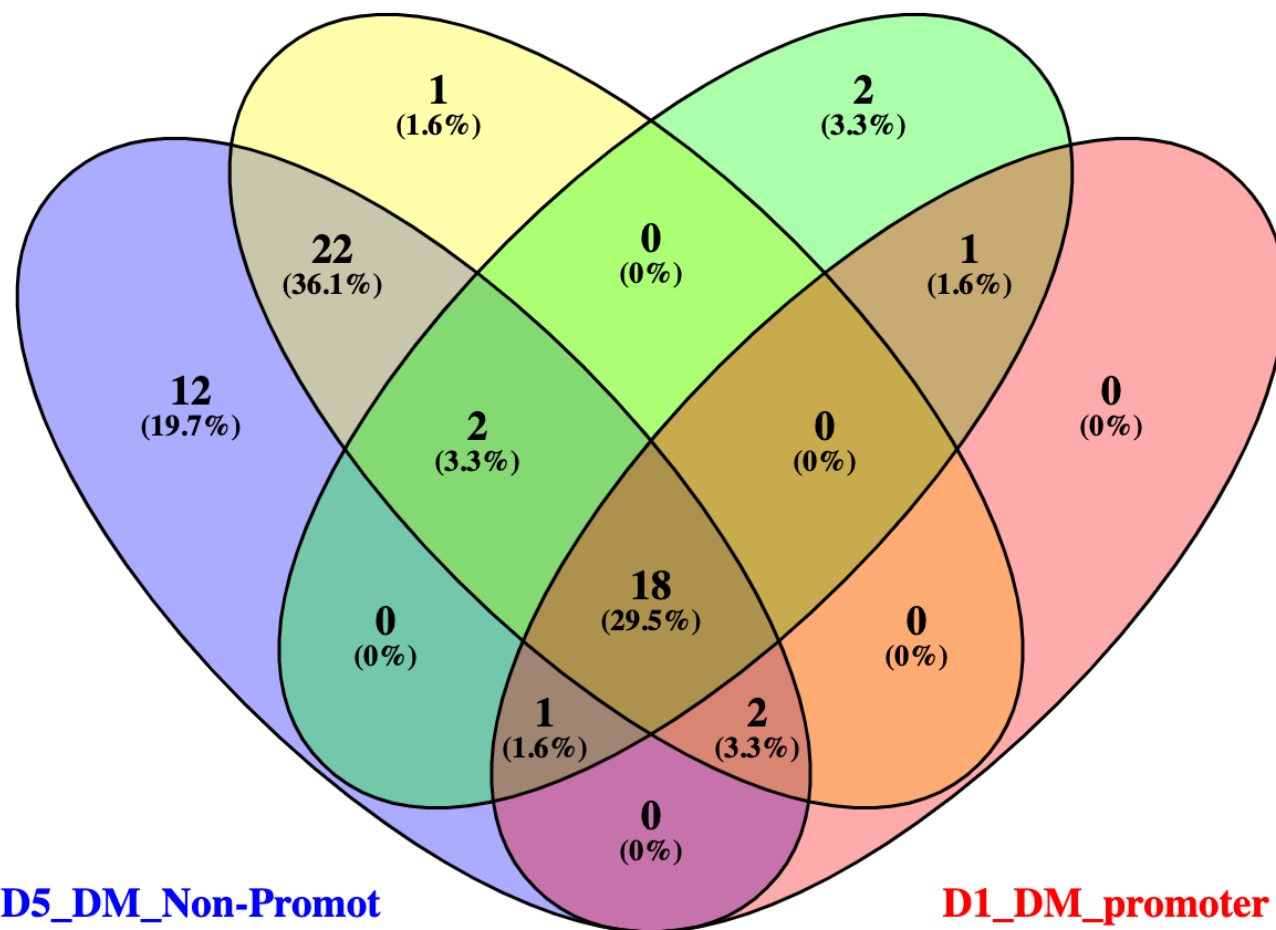

**D5\_DM\_Non-Promot**

**D1\_DM\_promoter**

Supplement: Supplementary file 1 [file cells-12-00501-s001.zip › Figure_S12.pdf]

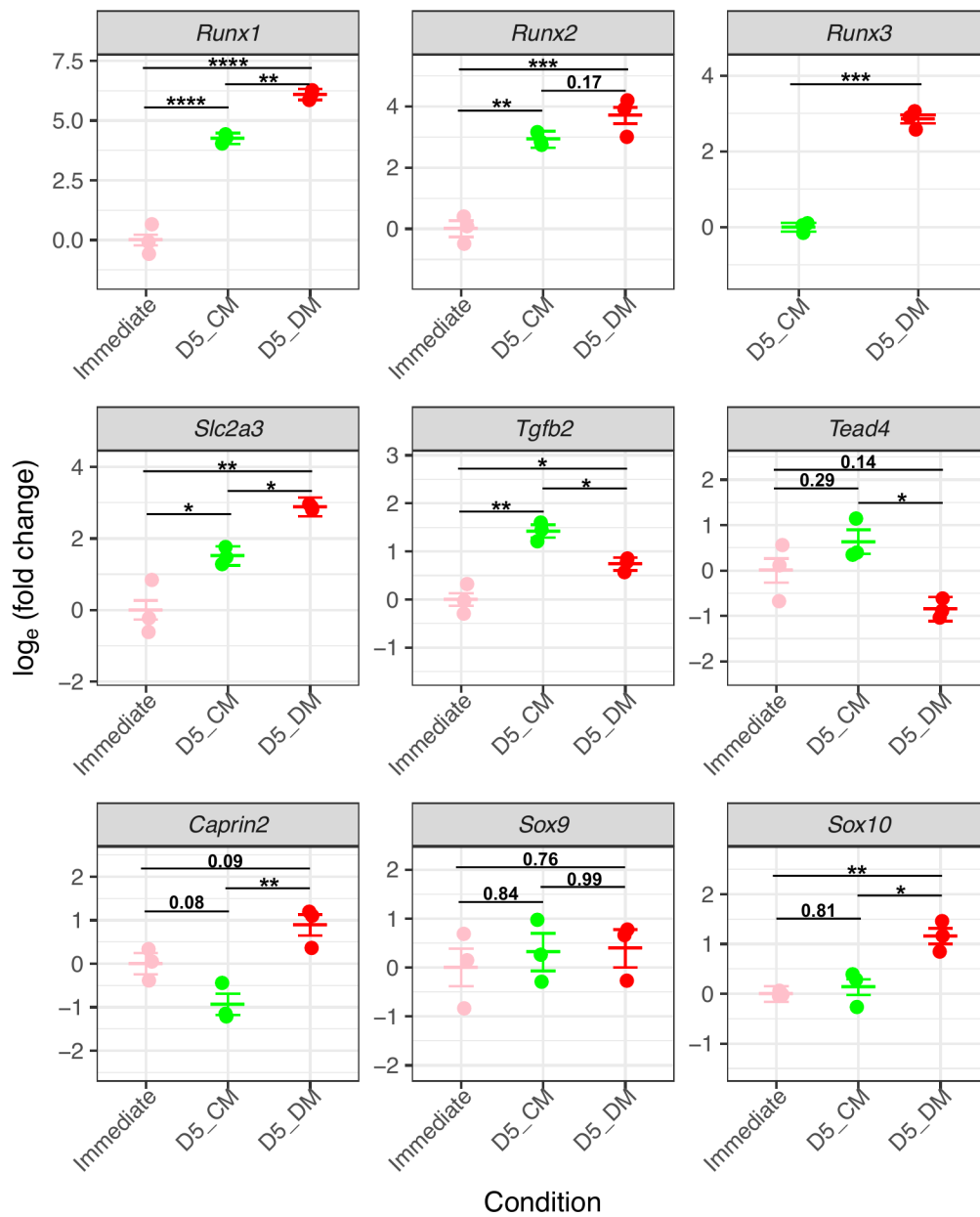

Supplement: Supplementary file 1 [file cells-12-00501-s001.zip › Figure_S13.pdf]

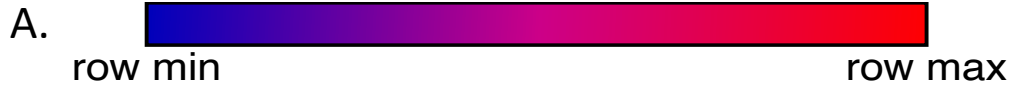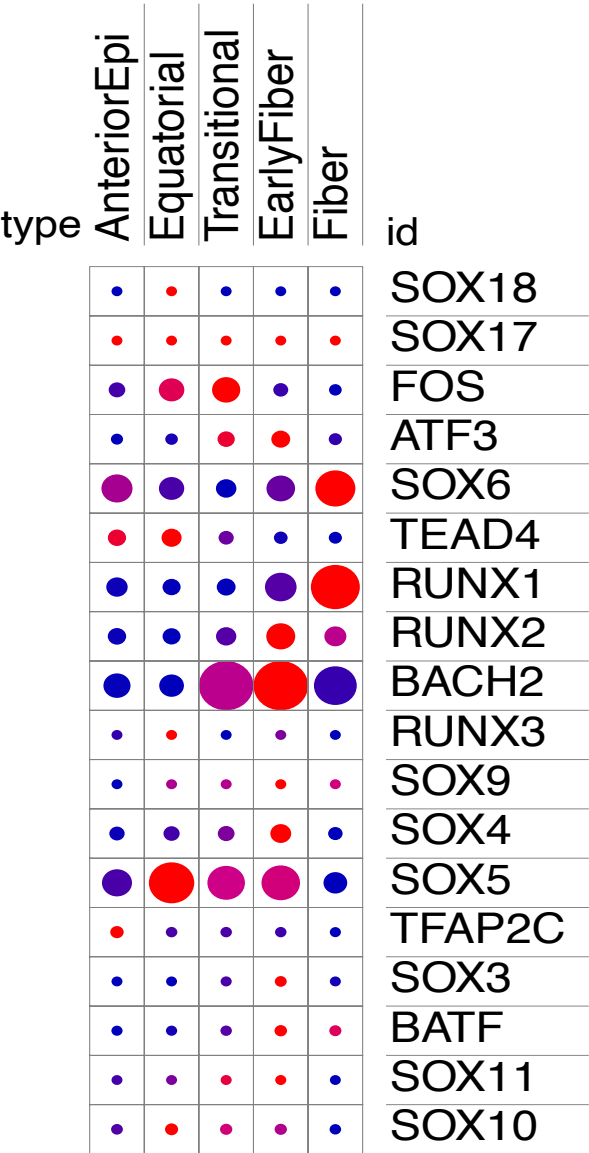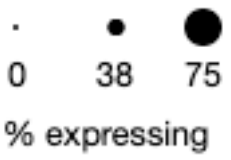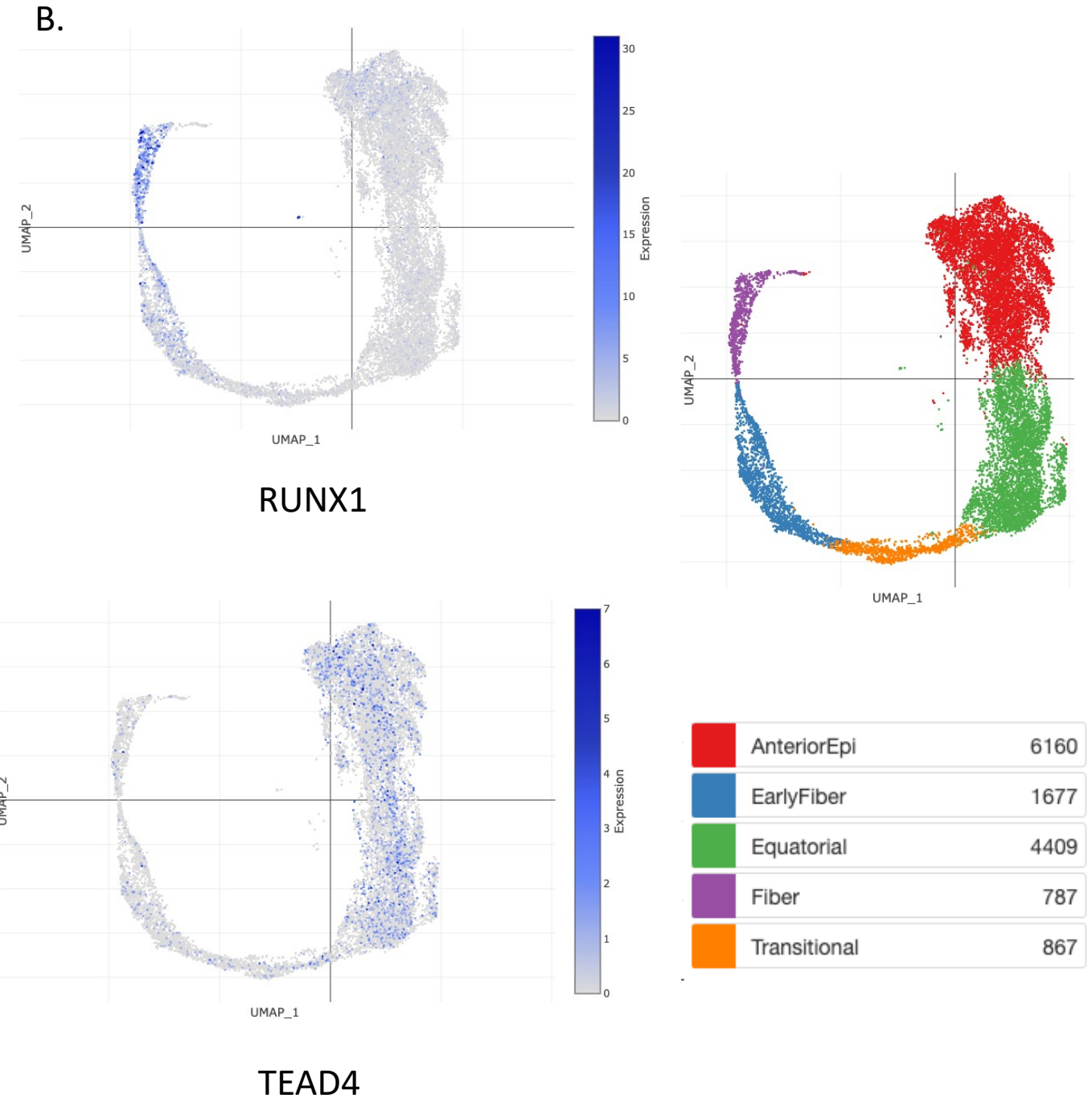

Supplement: Supplementary file 1 [file cells-12-00501-s001.zip › Figure_S14.pdf]

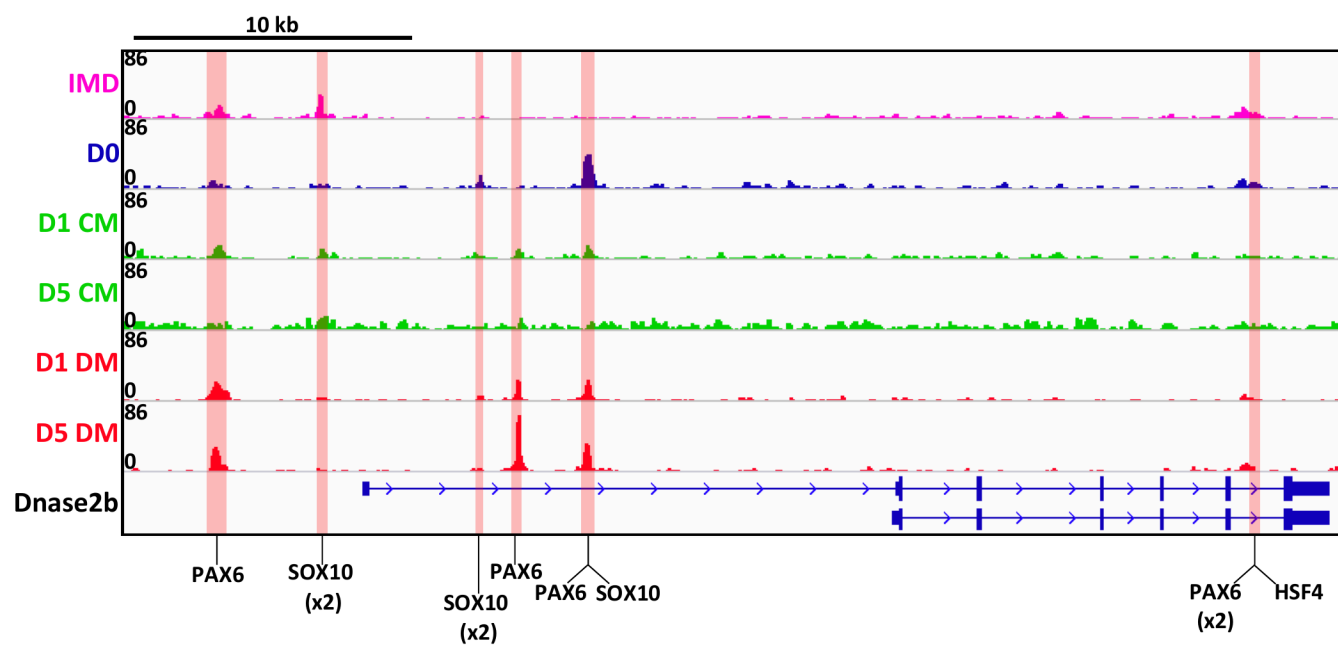

Supplement: Supplementary file 1 [file cells-12-00501-s001.zip › Figure_S15.pdf]

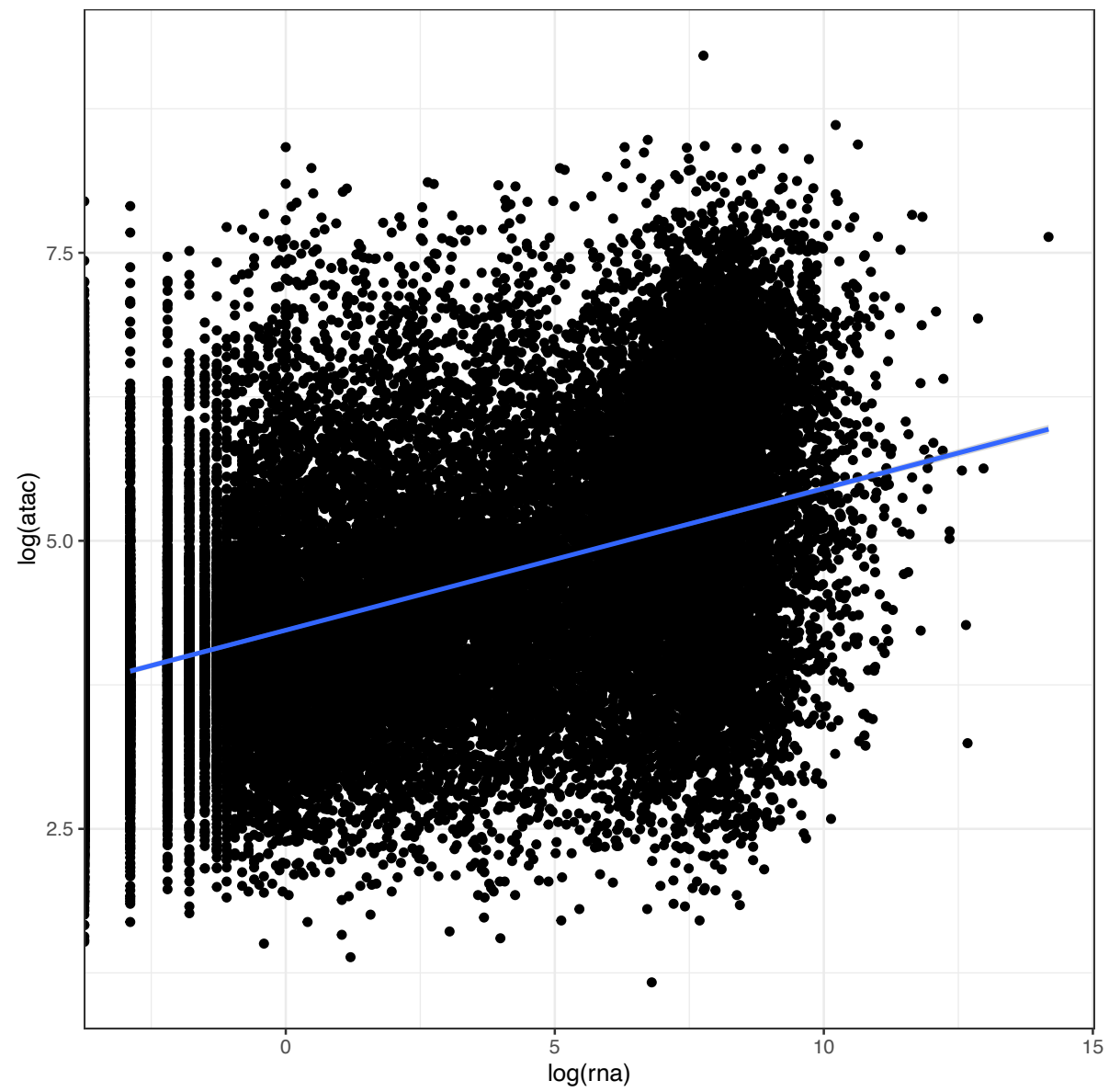

Supplement: Supplementary file 1 [file cells-12-00501-s001.zip › Figure_S2.pdf]

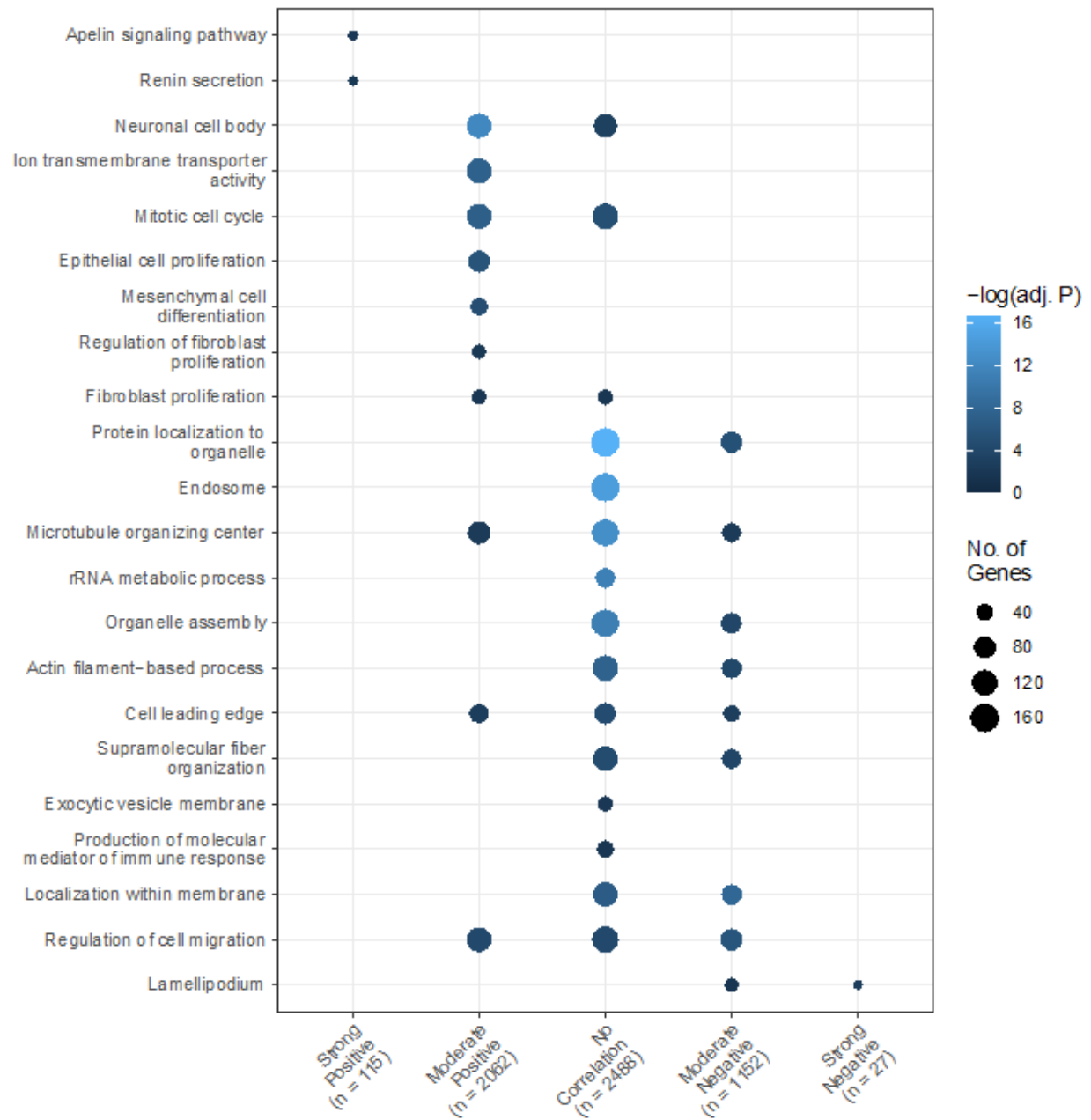

Supplement: Supplementary file 1 [file cells-12-00501-s001.zip › Figure_S3.pdf]

Feature Distribution

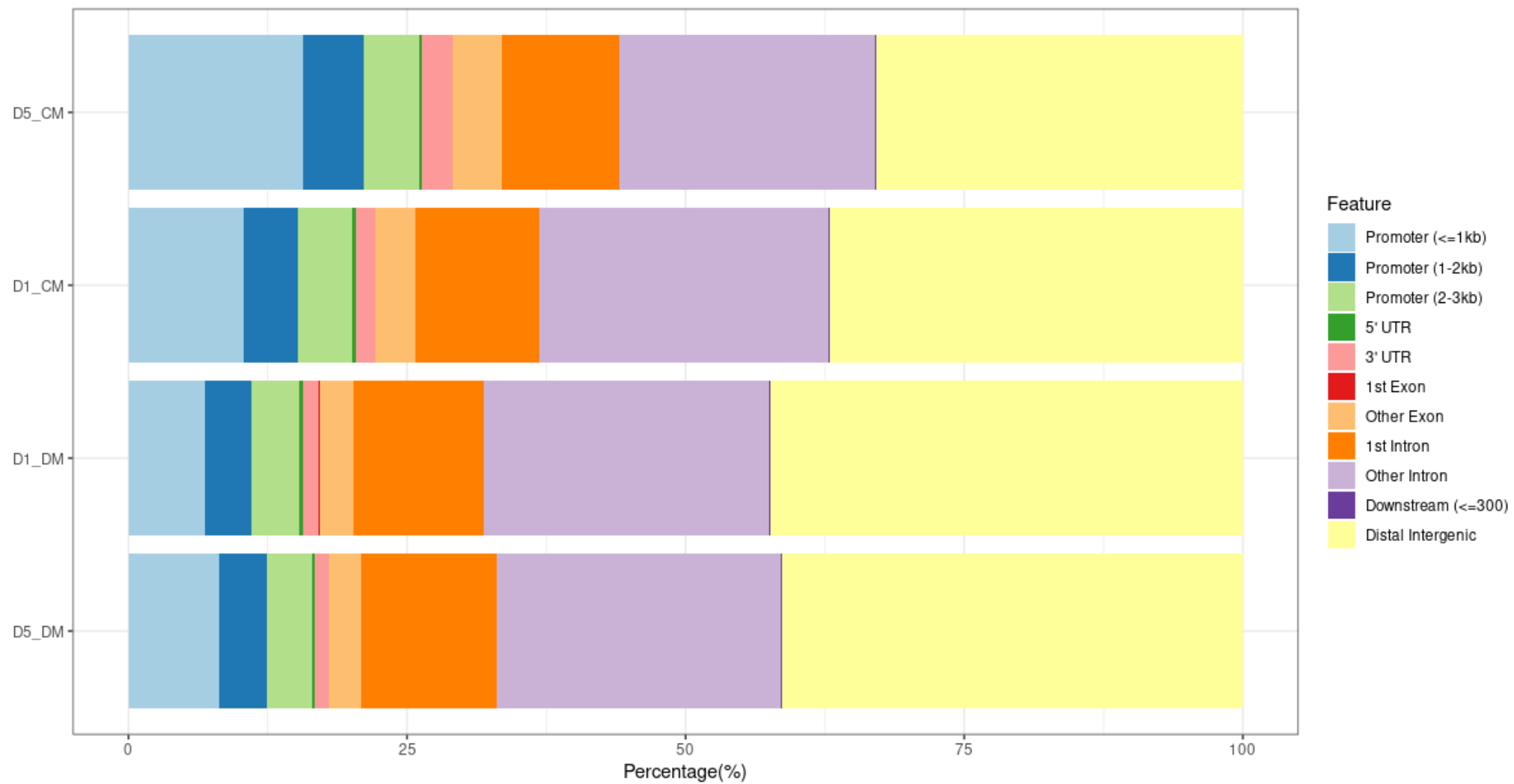

Supplement: Supplementary file 1 [file cells-12-00501-s001.zip › Figure_S4.pdf]

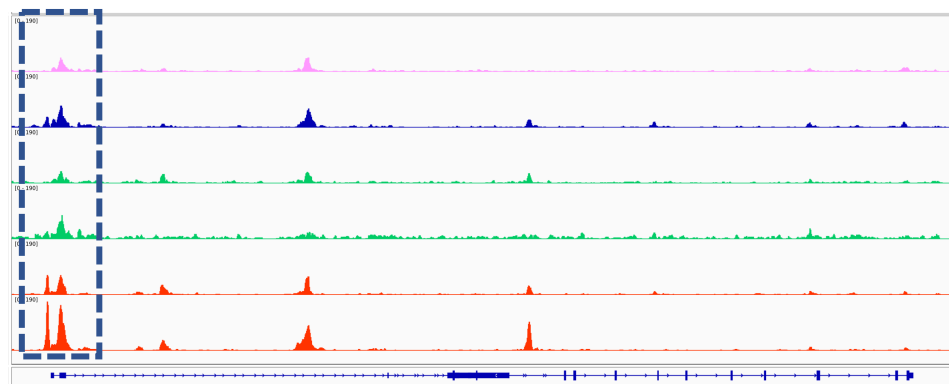

Pla2g7

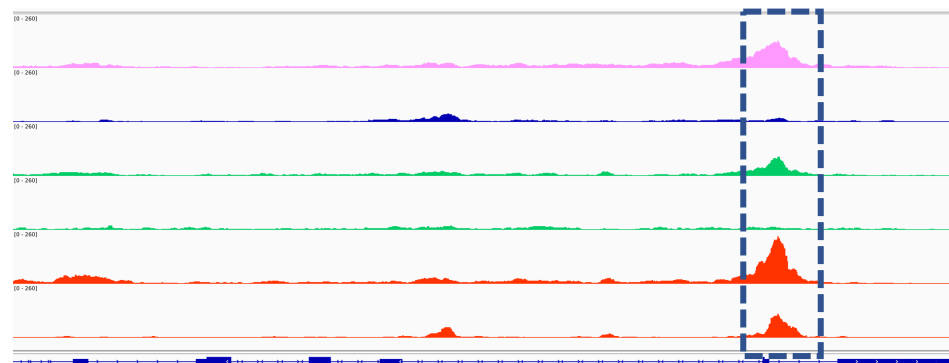

Crygc

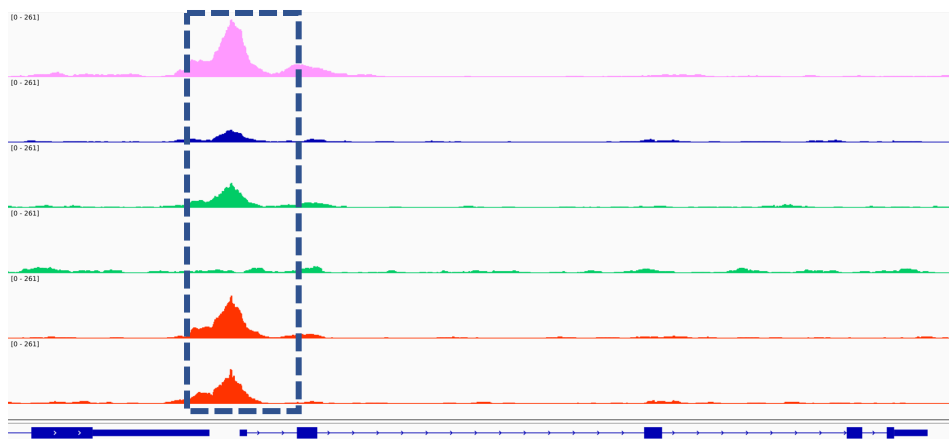

Lim2

IMD  
D0  
D1\_CM  
D5\_CM  
D1\_DM  
D5\_DM

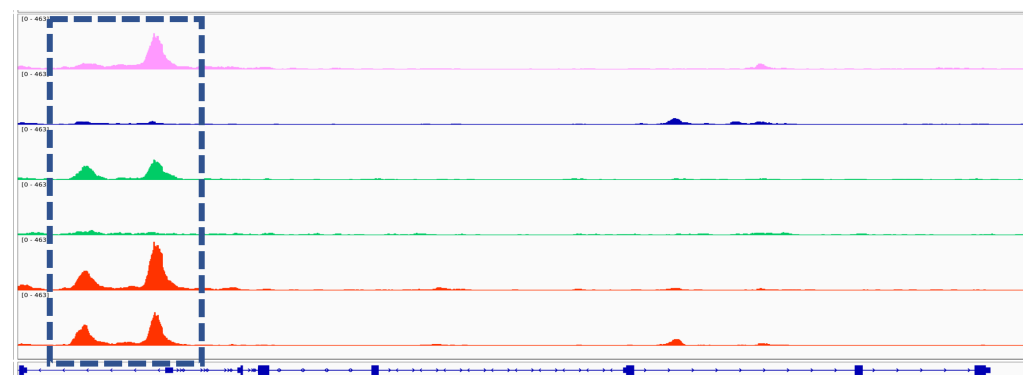

Cryba4/ Crybb1

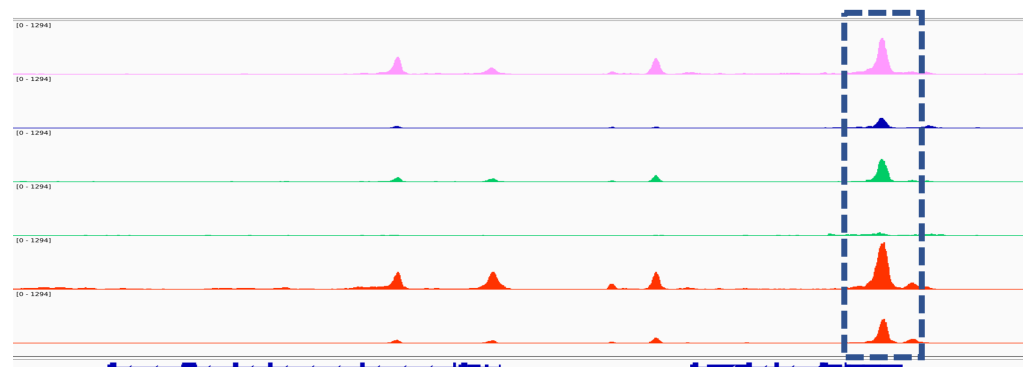

Crybb2/Crybb3

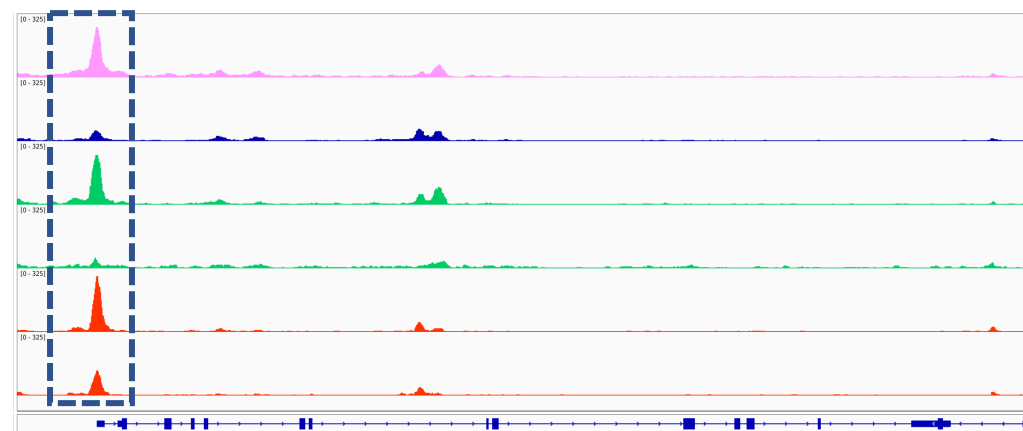

Lct1

IMD  
D0  
D1\_CM  
D5\_CM  
D1\_DM  
D5\_DM

Supplement: Supplementary file 1 [file cells-12-00501-s001.zip › Figure_S5.pdf]

A.

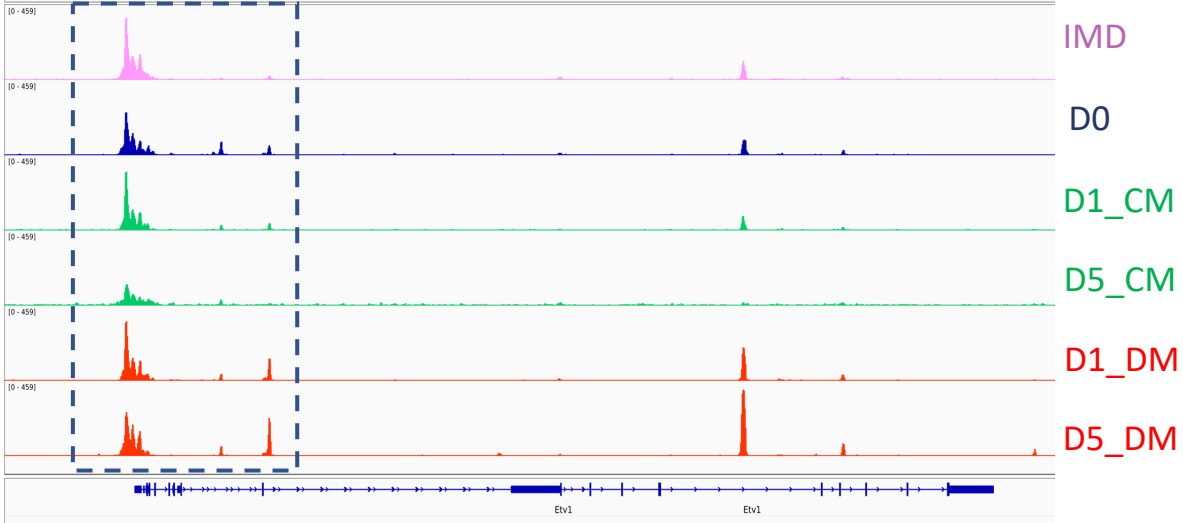

*Etv1*

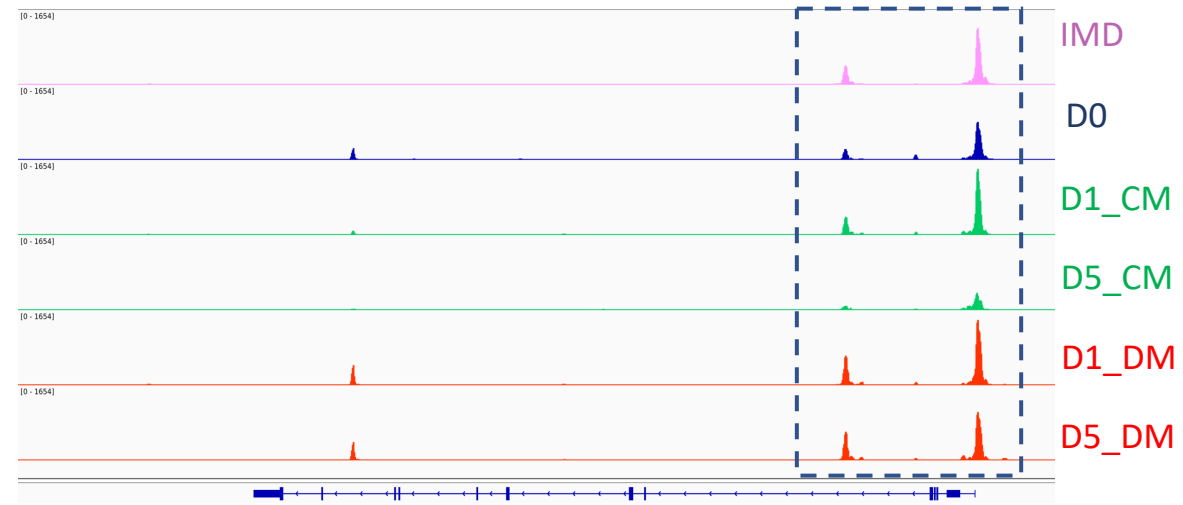

*Etv5*

B.

MAPK Signaling Genes

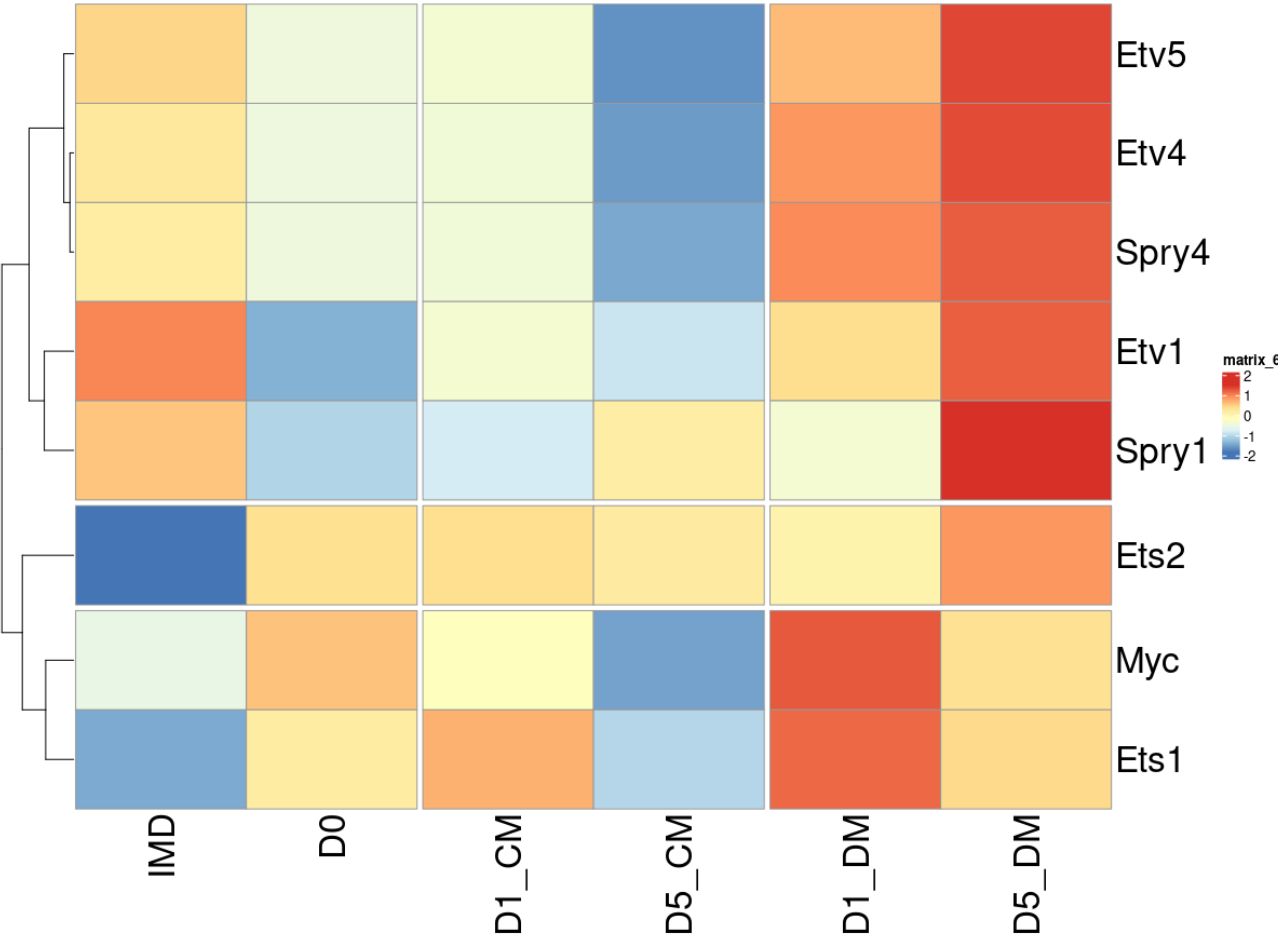

Supplement: Supplementary file 1 [file cells-12-00501-s001.zip › Figure_S6.pdf]

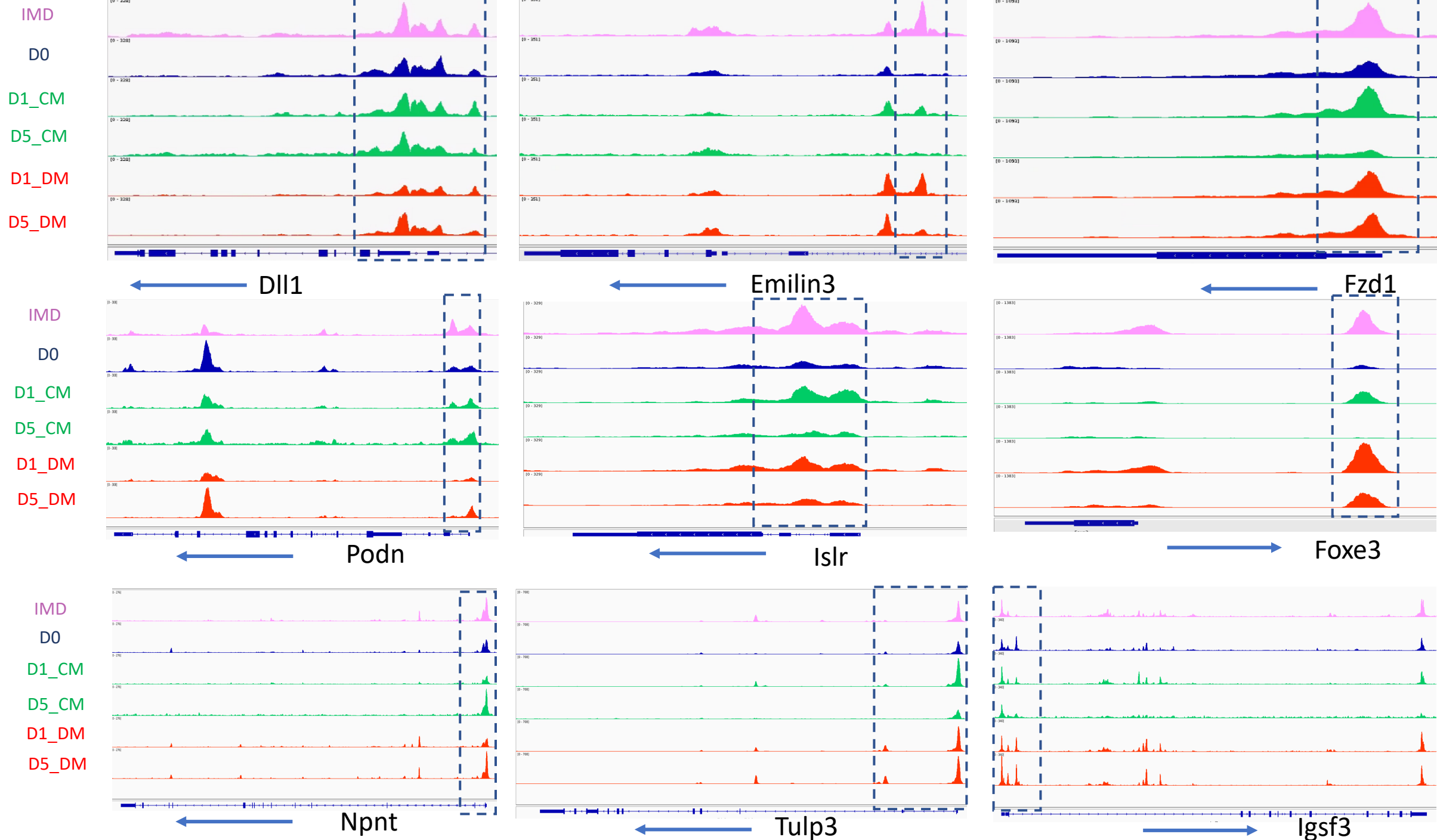

Supplement: Supplementary file 1 [file cells-12-00501-s001.zip › Figure_S7.pdf]

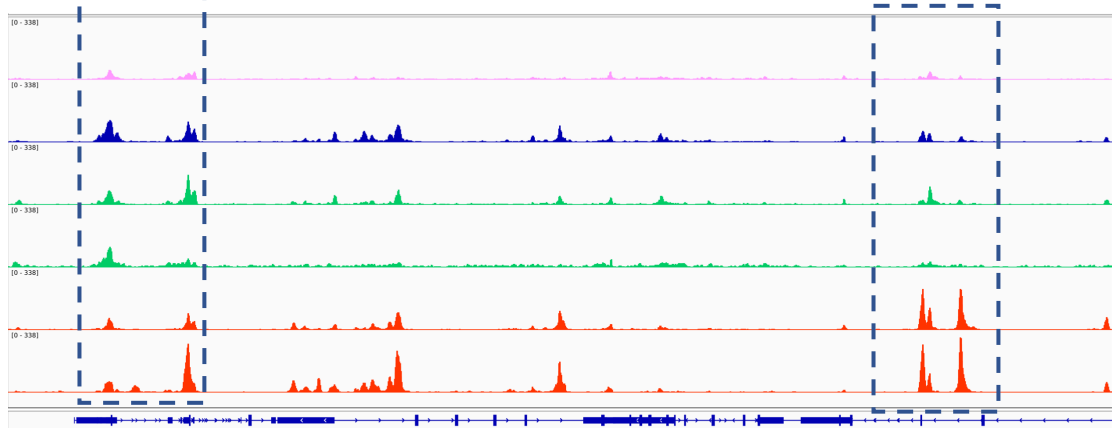

Ampd3

Rnf141

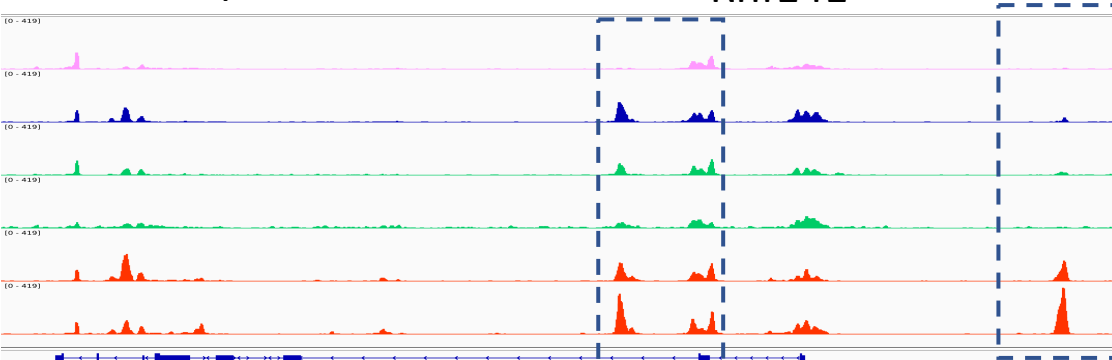

Lmo1

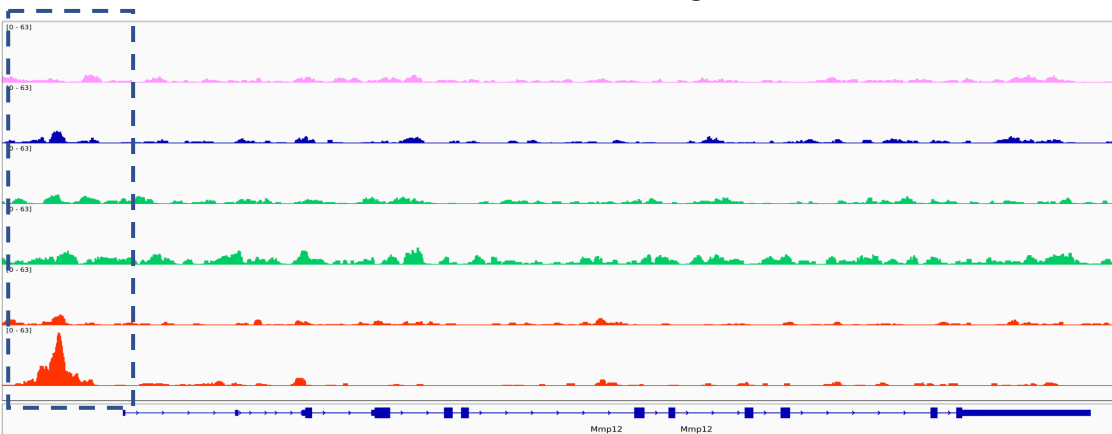

Mmp12

IMD  
D0  
D1\_CM  
D5\_CM  
D1\_DM  
D5\_DM

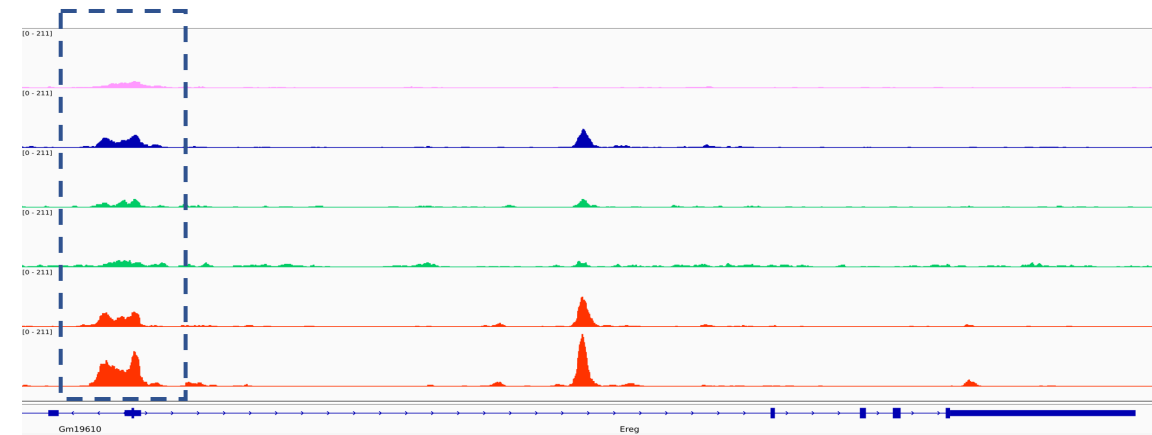

Ereg

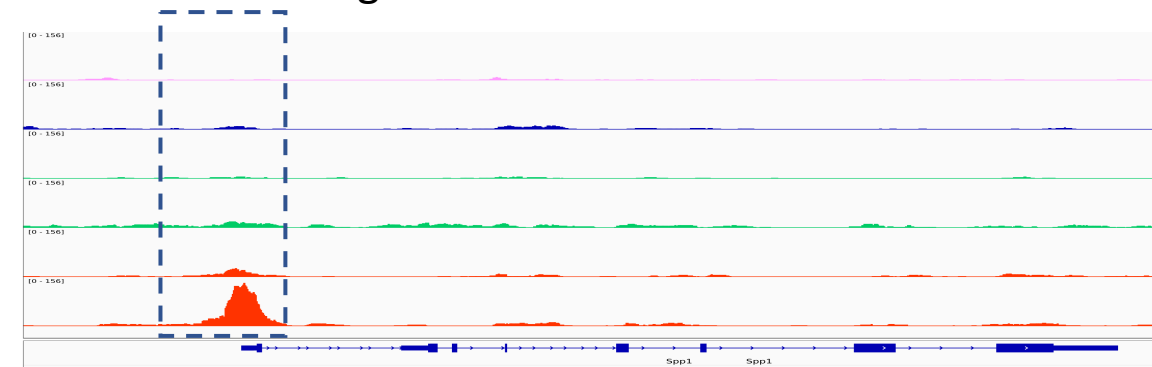

Spp1

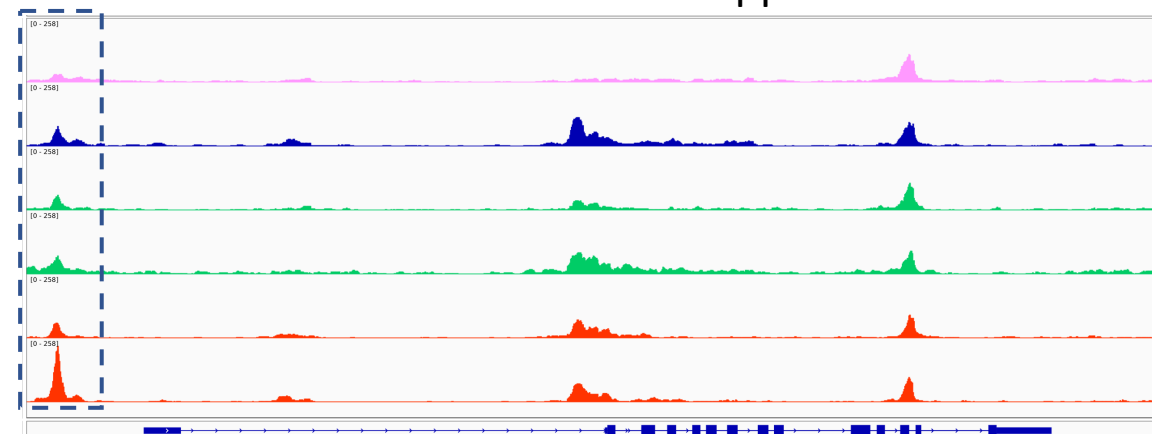

Mmp9

IMD  
D0  
D1\_CM  
D5\_CM  
D1\_DM  
D5\_DM

IMD  
D0  
D1\_CM  
D5\_CM  
D1\_DM  
D5\_DM

Supplement: Supplementary file 1 [file cells-12-00501-s001.zip › Figure_S8.pdf]

## ECM Organization Genes

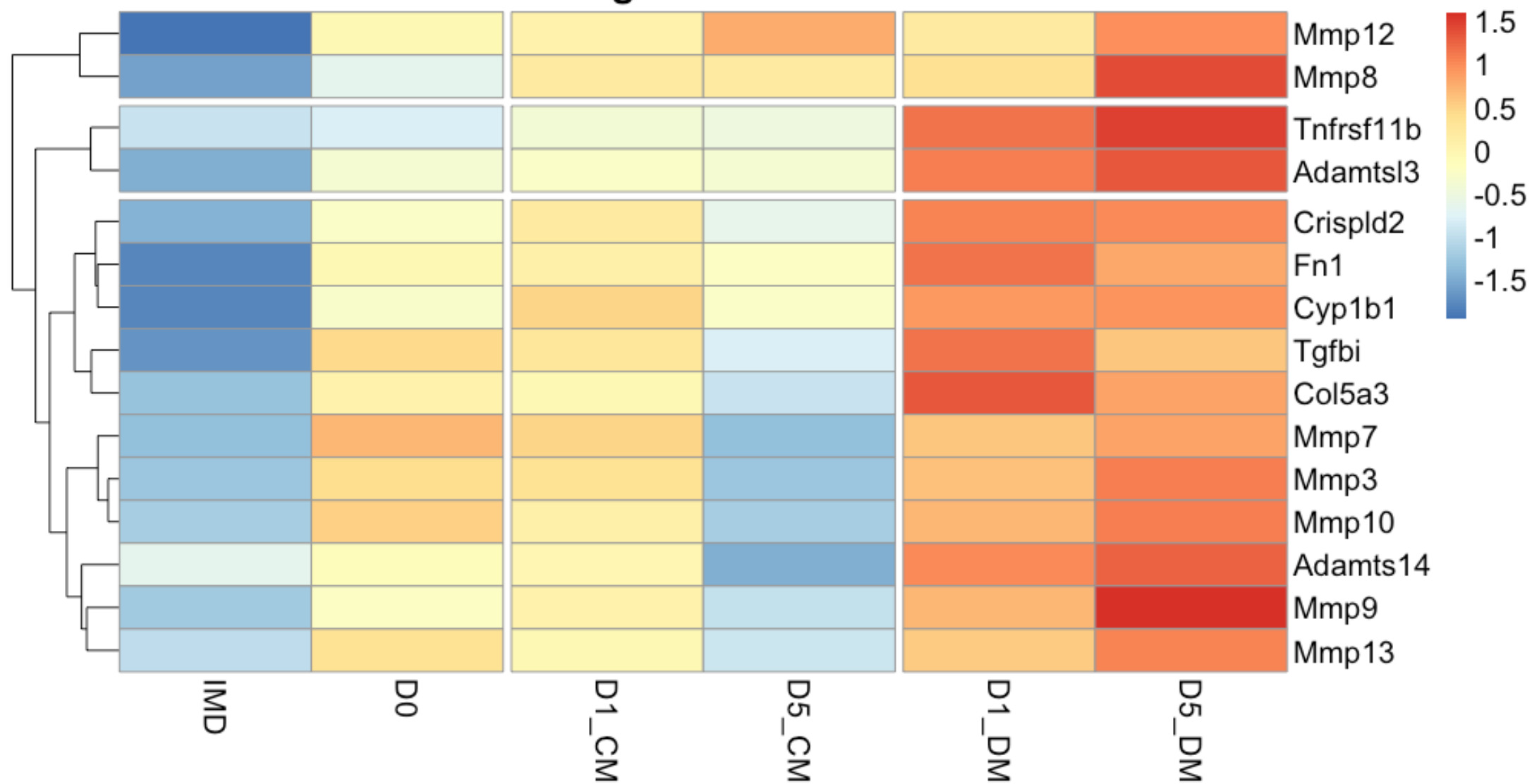

Supplement: Supplementary file 1 [file cells-12-00501-s001.zip › Figure_S9.pdf]
